# Obesity-related hypertension: Findings from The Korea National Health and Nutrition Examination Survey 2008–2010

**DOI:** 10.1371/journal.pone.0230616

**Published:** 2020-04-21

**Authors:** Hong Seok Lee, Yong-Moon Park, Kyungdo Han, Jin-Hong Yang, Seungwon Lee, Seong‐Su Lee, Soonjib Yoo, Sung Rae Kim

**Affiliations:** 1 Division of Cardiology, Department of Medicine, Mayo Clinic, Scottsdale, AZ, United States of America; 2 Division of Cardiology, Department of Medicine, University of California Riverside, Riverside, CA, United States of America; 3 Johns Hopkins Bloomberg School of Public Health, Baltimore, MD, United States of America; 4 Epidemiology Branch, National Institute of Environmental Health Sciences, National Institutes of Health, Research Triangle Park, NC, United States of America; 5 Department of Statistics and Actuarial Science, Soongsil University, Seoul, Korea; 6 Department of Emergency Medicine, College of Medicine, The Catholic University of Korea, Seoul, Korea; 7 Department of Anesthesiology and Pain Medicine, Samsung Medical Center, Sungkyunkwan University School of Medicine, Seoul, Korea; 8 Division of Endocrinology and Metabolism, Department of Internal Medicine, Bucheon St. Mary’s Hospital, College of Medicine, The Catholic University of Korea, Seoul, Korea; McMaster University, CANADA

## Abstract

We aimed to investigate the association of various obesity parameters and phenotypes with hypertension in nationally representative Korean adults. Among adults aged 19 years and older who participated in the Korea National Health and Nutrition Examination Survey in 2008–2010, a total of 16,363 subjects (8,184 men and 8,179 women) were analyzed. Hypertension was defined as blood pressure of 140/90 mm Hg or higher or taking antihypertensive medication. Multiple logistic regression analysis was used to estimate multivariable-adjusted odds ratios (ORs) and 95% confidence intervals (CIs). Higher obesity parameters [body mass index (BMI) representing general obesity, waist circumference (WC) representing central obesity, and percentage body fat (PBF) representing elevated body fat] were consistently associated with increased odds of prevalent hypertension (OR, 7.54; 95% CI, 5.89–9.65 for BMI ≥30 vs. 18.5–23; OR, 3.97; 95% CI, 3.41–4.63 for WC ≥95 cm in males and ≥90 cm in females vs. <85 cm in males and <80 cm in females; OR, 3.56; 95% CI, 3.05–4.15 for PBF, highest vs. lowest quartile; all *p* trends<0.0001). These associations were stronger in the younger age group (<40 years), and were observed in both sexes. Furthermore, even in individuals with normal BMI (18.5–23), the odds of prevalent hypertension were consistently increased in those with central obesity (WC≥90 cm in males, WC≥80 cm in females; normal weight central obesity phenotype) (OR, 1.89; 95% CI, 1.63–2.19) and those with high PBF (highest quartile of PBF; normal weight obesity phenotype) (OR, 1.49; 95% CI, 1.25–1.77). These associations were consistent with updated hypertension guidelines in 2017. Obesity may be positively associated with hypertension, regardless of obesity parameters. Even within normal BMI range, high WC and high PBF may be associated with hypertension.

## Introduction

The increasing prevalence of obesity worldwide represents a major public health problem. Obesity reduces healthy life years, increases mortality, and poses an increasing health economic burden [1,2]. Obesity increases the risk of metabolic diseases, such as hypertension, diabetes, and dyslipidemia, which lead to increases in cardiovascular morbidity and mortality [3,4]. Obesity can be represented by various measurements, such as body mass index (BMI) [5] waist circumference (WC) [6], and percentage body fat (PBF) [7].

Hypertension prevalence in obese people with high BMI was reported to be 40.5% in the US [8]. The prevalence of hypertension has been reported to increase progressively with increasing BMI [9]. There is evidence to indicate that weight gain may increase blood pressure (BP) [10], and that weight reduction can prevent or delay obesity-related risk factors for cardiovascular diseases, including hypertension. It has been shown that increased BF composition is associated with hypertension [11]. Abnormalities in body fat distribution also might play an important role in the development of hypertension [12,13]. Central body fat has been associated with insulin resistance and dyslipidemia, suggesting that it is a more potent determinant of BP elevation than peripheral body fat. Visceral obesity appears to be especially important in the activation of the sympathetic nervous system and the renin-angiotensin-aldosterone system, thereby increasing the risk for the development of hypertension and its associated comorbidities. [14,15]

Several epidemiologic studies have explored hypertension prevalence in obese patients defined by BMI [3,16–20]. However, it is not known whether the association between various obesity parameters and hypertension differs by specific obesity phenotypes, such as normal weight obesity (represented by normal BMI with high PBF) and normal weight central obesity phenotypes (represented by normal BMI with high WC). Therefore, we aimed to investigate the relationship of various obesity parameters and phenotypes with hypertension using a nationally representative sample in Korea.

## Materials and methods

### Study population

This was a cross-sectional study using a nationally representative survey performed by the Korea Centers for Disease Control and Prevention. Data between 2008 and 2010 of the Korea National Health and Nutrition Examination Survey (KNHANES) was obtained for analysis. KNHANES adopted a rolling survey sampling design that applied a complex, stratified, multistage probability-cluster survey using a representative Korean population sample aged 1 year and above. In the present analysis, among the subjects aged 19 years and older who completed both a health interview survey and a health examination survey from 2008 to 2010, a total of 16,363 subjects (8,184 men and 8,179 women) were included in this study. The KNHANES was conducted by specially trained interviewers or examiners who were not provided with any prior information about the participants. The survey was composed of three parts: a health interview survey, a health examination survey, and a nutrition survey. Extra details regarding the study design and methods are provided elsewhere. All of the participants signed an informed consent in this survey. This study was approved by the Institutional Review Board of the Catholic University of Korea. We confirm that all research was performed in accordance with relevant guidelines/regulations, and informed consent was obtained from all participants and/or their legal guardians.

### Measurements

All subject measurements were performed by trained examiners. Information regarding the demographic status and health-related characteristics was collected during the health interview survey and included the following: age, educational attainment, household income, living with a spouse or not, and use of antihypertensive medication. In addition, lifestyle characteristics including smoking, alcohol consumption, and exercise were investigated using self-administered questionnaires. The education level was split into three groups: 6 years or fewer (elementary school or less), 7 to 12 years (middle school to high school), and 13 years or more (college or higher). Household income was divided into quartiles, and the lowest quartile was the lowest income class. Smoking status was categorized into three groups: current smoker, never smoked, or past smoker. Alcohol consumption status was classified into three groups: non-drinker, mild-to-moderate drinker (< 30.0 g alcohol/day), and heavy drinker (≥ 30.0 g alcohol/day), after converting the average frequency and amount of alcoholic beverages into the amount of pure alcohol (in grams) consumed per day. Regular exercise was designated as ‘yes’ when the subject reported moderate exercise for more than 30 minutes at a time and more than five times per week or vigorous exercise for more than 20 minutes at a time and more than three times per week on a regular basis. Nutrient intakes, including total energy and sodium consumptions, were assessed with a 24-hour dietary recall questionnaire administered by a trained dietician. Height was measured to the nearest 0.1 cm by a portable stadiometer (SECA 225, SECA, Deutschland, Hamburg, Germany) while the subjects were standing barefoot. Body weight was measured to the nearest 0.1 kg on a balanced scale (GL-6000-20, CAS KOREA, Seoul, Korea) while the participants wore a lightweight gown. BMI was calculated from the measured height and weight using the following formula: BMI = weight (kg) / height squared (m^2^). We used BMI classification for the Asian-Pacific region [21]. BMI was categorized as follows: normal weight (18.5 ≤ BMI < 23 kg/m^2^), overweight (23 ≤ BMI < 25 kg/m^2^), Obese I (25 ≤ BMI < 30 kg/m^2^), and Obese II (BMI ≥ 30 kg/m^2^). WC was measured to the nearest 0.1 cm using a measuring tape (SECA 200, SECA, Deutschland) in a horizontal plane at the level of the midpoint between the iliac crest and the costal margin at the end of a normal expiration. The PBF (fat mass/total mass) was measured using dual-energy X-ray absorptiometry (DXA; QDR 4500A, Hologic Inc., Waltham, MA, US) [22] and was divided into four even quartiles. BP was measured from the right arm using a standard mercury sphygmomanometer (Baumanometer, WA Baum Co., New York, USA) after 5 minutes of rest in the sitting position. Systolic and diastolic BPs were measured three times at 30-second intervals, and the second and third measurements were averaged to produce the final BP used for analysis.

### Definitions

Hypertension was defined as an average BP of 140/90 mm Hg or higher, or if the participant was taking antihypertensive medication. Normotension was defined as BP less than 120/80 mm Hg. Prehypertension is the status of systolic BP of 120 to 139 mm Hg or diastolic BP 80 to 89 mm Hg based on the Seventh Report of the Joint National Committee on Prevention, Detection, Evaluation and Treatment of High Blood Pressure (JNC 7) guideline [23]. A new guideline, proposed by the American College of Cardiology and the American Heart Association in 2017, set to lower the definition of hypertension to systolic BP of 130 mm Hg or higher or diastolic BP of 80 mm Hg or higher, [24] was used to conduct a sensitivity analysis. In addition, for abdominal obesity, we used sex-specific cutoff points individualized for men and women: 90 cm for men, and 80 cm for women^21^. The normal weight obesity phenotype was defined when participants had normal body weight by BMI, but had a high PBF (highest quartile of PBF) [25], whereas the normal-weight central obesity phenotype was defined when participants had normal body weight, but had high WC (≥90 cm for men and ≥80 cm for women) [26]. Participants were considered to have diabetes if fasting plasma glucose was 126 mg/dL or greater or they were receiving insulin or oral diabetes medications. Participants were considered to have hypercholesterolemia if their total cholesterol was 240 mg/dL or greater or if they were taking cholesterol-lowering medication.

### Statistical analysis

All statistical analyses were performed with SAS, version 9.3 (SAS Institute, Inc., Cary, North Carolina) for the complex KNHANES sampling design. Subjects’ characteristics were shown as mean with standard error (SE) for continuous variables and percentage with SE for categorical variables. Continuous variables were compared using linear regression analysis, and categorical data were compared using the Rao-Scott chi-square test, which is a design-adjusted Pearson chi-square test used for complex survey data. For subgroup analyses, the domain option was used to preserve appropriate subsamples in the complex sampling designs.

We estimated odds ratios (ORs) and 95% confidence intervals (CIs) for the association between obesity parameters and prevalent hypertension using multiple logistic regression. Tests for linear trend across each category of obesity parameter were performed by modeling an ordinal variable for each obesity category. Potential confounders or effect modifiers were ascertained a priori based on a literature review. The following covariates were included in multivariable-adjusted models: age and sex in model 1; and age, sex, smoking (never smoked, current smoker, past smoker), alcohol consumption (non-drinker, mild-to-moderate drinker, heavy drinker), physical activity (regular exercise, non-regular exercise, no exercise), living with spouse or not, income (quartiles), educational attainment (≤ 6 years, 7–12 years, ≥13 years), energy intake from fat, and sodium consumption in model 2. We evaluated whether there is a synergistic interaction between BMI and WC as well as BMI and PBF in relation to prevalent hypertension. We also assessed potential effect modification by diabetes, education, income, smoking habit, alcohol consumption, and exercise through stratified analysis and interaction testing using a likelihood ratio test. Furthermore, we examined the associations by sex to explore sex-specific association between obesity parameters and hypertension. Statistical significance was determined with two-sided tests. Significance level α was set at 0.05.

## Results

Differences in general characteristics by BP classification are summarized in Table 1. Hypertensive participants were relatively older (*p* < 0.001) compared with those without hypertension or pre-hypertension, were more likely to have past smoking history (*P*<0.001), and were less likely to be mild-moderate drinkers (*P*<0.001). They were also more likely to have lower educational level and household income (both *P*<0.001); to have higher levels of BMI, WC, and PBF (*P*<0.001); and to have diabetes, hypercholesterolemia, and lower income (*P*<0.001). They were less likely to consume dietary fats but were more likely to consume sodium.

**Table 1:** General characteristics of study population according to blood pressure classification.

	Blood Pressure Classification			
	Normotension	Prehypertension	Hypertension	*p*
Characteristic	(n = 7112)	(n = 4028)	(n = 5223)	
Age (year)				<0.001
19–39	57.7(0.9)	39.8(1.2)	14.0(0.8)	
40–64	37.8(0.8)	48.3(1.1)	56.1(0.9)	
≥65	4.5(0.3)	11.9(0.6)	30.0(0.9)	
Male sex	40(0.7)	60.3(1.0)	56.2(0.8)	<0.001
Smoking				<0.001
Never	62.3(0.7)	49.7(1.0)	51.6(0.8)	
Past	12(0.5)	19.4(0.7)	23.7(0.7)	
Current	25.7(0.7)	30.9(1.0)	24.7(0.8)	
Alcohol consumption				<0.001
Non-drinker	21.1(0.6)	21.3(0.7)	31.3(0.8)	
Mild to moderate drinker	71.4(0.7)	66.7(0.9)	55.5(0.9)	
Heavy drinker	7.5(0.4)	12(0.6)	13.2(0.6)	
Regular physical activity	24.7(0.6)	25.9(0.9)	25.6(0.9)	0.470
Living with a spouse	64.9(1)	69.2(1.1)	73(0.8)	<0.001
Household income				<0.001
Lowest quartile	11.9(0.6)	14.9(0.8)	25.6(0.9)	
Medium	57.7(1)	54.8(1.3)	49.8(1.1)	
Highest quartile	30.4(1)	30.3(1.3)	24.6(1)	
Educational attainment (year)				<0.001
Elementary school or less (≤6)	9.4(0.5)	19.4(0.9)	37.8(1)	
Middle to high school (7–12)	53.1(0.9)	49.9(1.0)	42.7(0.9)	
College or higher (≥13)	37.5(0.9)	30.(1.1)	19.6(1)	
BMI (kg/m^2^)	22.6±0	23.9±0.1	25±0.1	<0.001
WC (cm)	77.2±0.2	81.9±0.2	85.7±0.2	<0.001
Percentage body fat (%)	27.3±0.2	26.6±0.2	28.6±0.2	<0.001
SBP (mmHg)	105.1±0.2	122±0.2	136.1±0.3	<0.001
DBP (mmHg)	69.1±0.1	80.8±0.1	86.9±0.2	<0.001
Total energy intake (kcal)	1967.2±15.4	2068.1±21.6	1938±17.4	<0.001
Energy intake from fat (%)	19±0.2	17.6±0.2	15.4±0.2	<0.001
Sodium	4997.7±62.4	5013.6±57.7	5436.3±74.2	< .001
Diabetes mellitus	3.3(0.3)	7.1(0.5)	17.3(0.6)	<0.001
Hypercholesterolemia	5.6(0.3)	10.1(0.6)	19.8(0.7)	<0.001

Data are shown as means ± standard error (SE) or percentages (SE).

Abbreviations: SBP; Systolic Blood Pressure, DBP; Diastolic Blood Pressure, BMI; Body Mass Index, WC; Waist Circumference.

**Fig 1:**
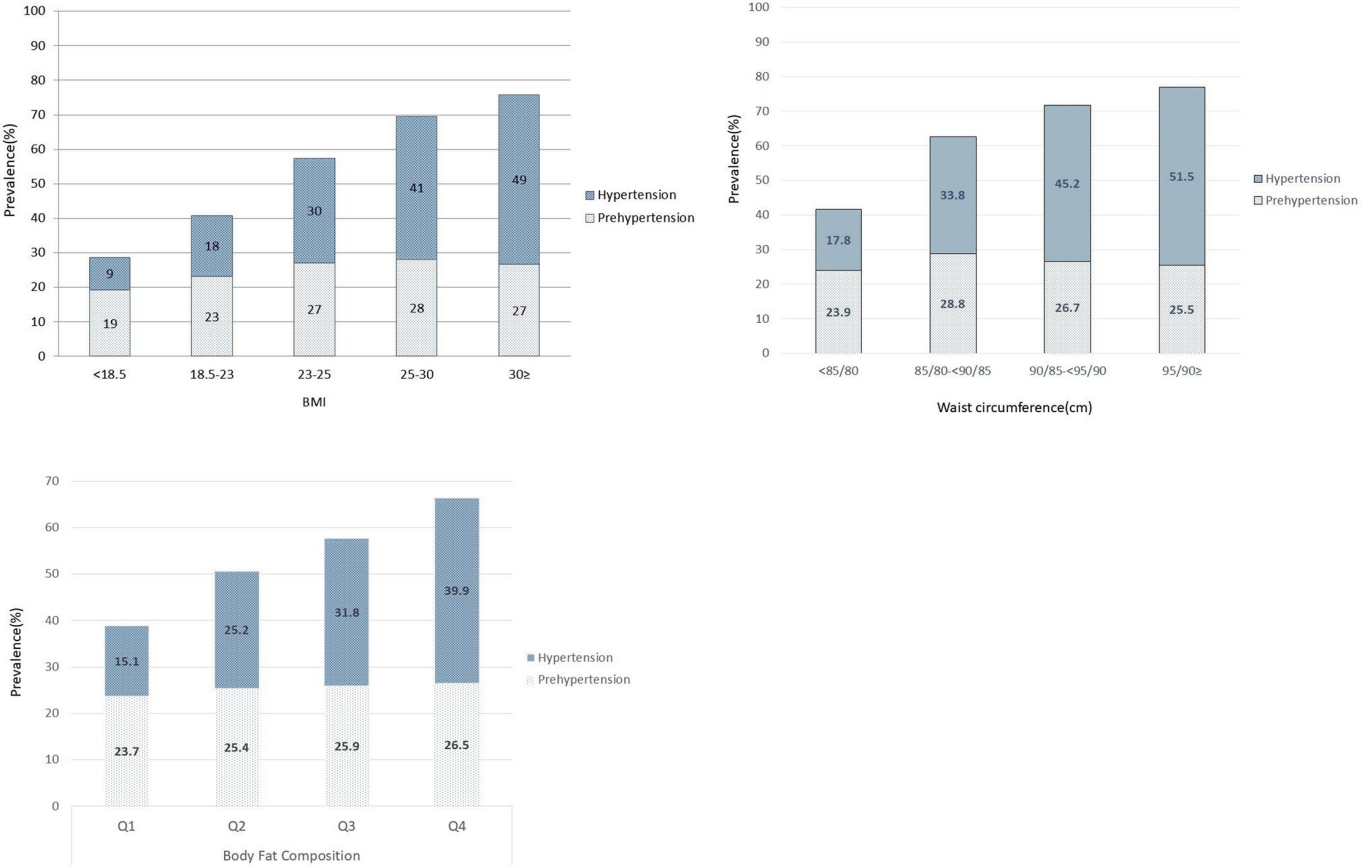
**a.** Prevalence of hypertension and prehypertension with increasing body mass index (BMI, kg/m^2^) categories. **b.** Prevalence of hypertension and prehypertension with increasing waist circumference categories. **c.** Prevalence of hypertension and prehypertension with increasing quartiles (Q1 through Q4) of percentage body fat. Abbreviations: <85/80: waist circumference less than 85 cm in males and less than 80 cm in females; 85/80–<90/85, waist circumference between 85 and 90 cm in males and between 80 and 85 cm in females; 90/85–95/90, waist circumference between 90 and 95 cm in males and between 85 and 90 cm in females; ≥95/90: waist circumference more than 95 cm in males and more than 90 cm in females. Legend: Prevalence of hypertension was increased with increasing BMI, waist circumference, and increasing quartiles of percentage of body fat *(all p* trends <0.001).

**Fig 2:**
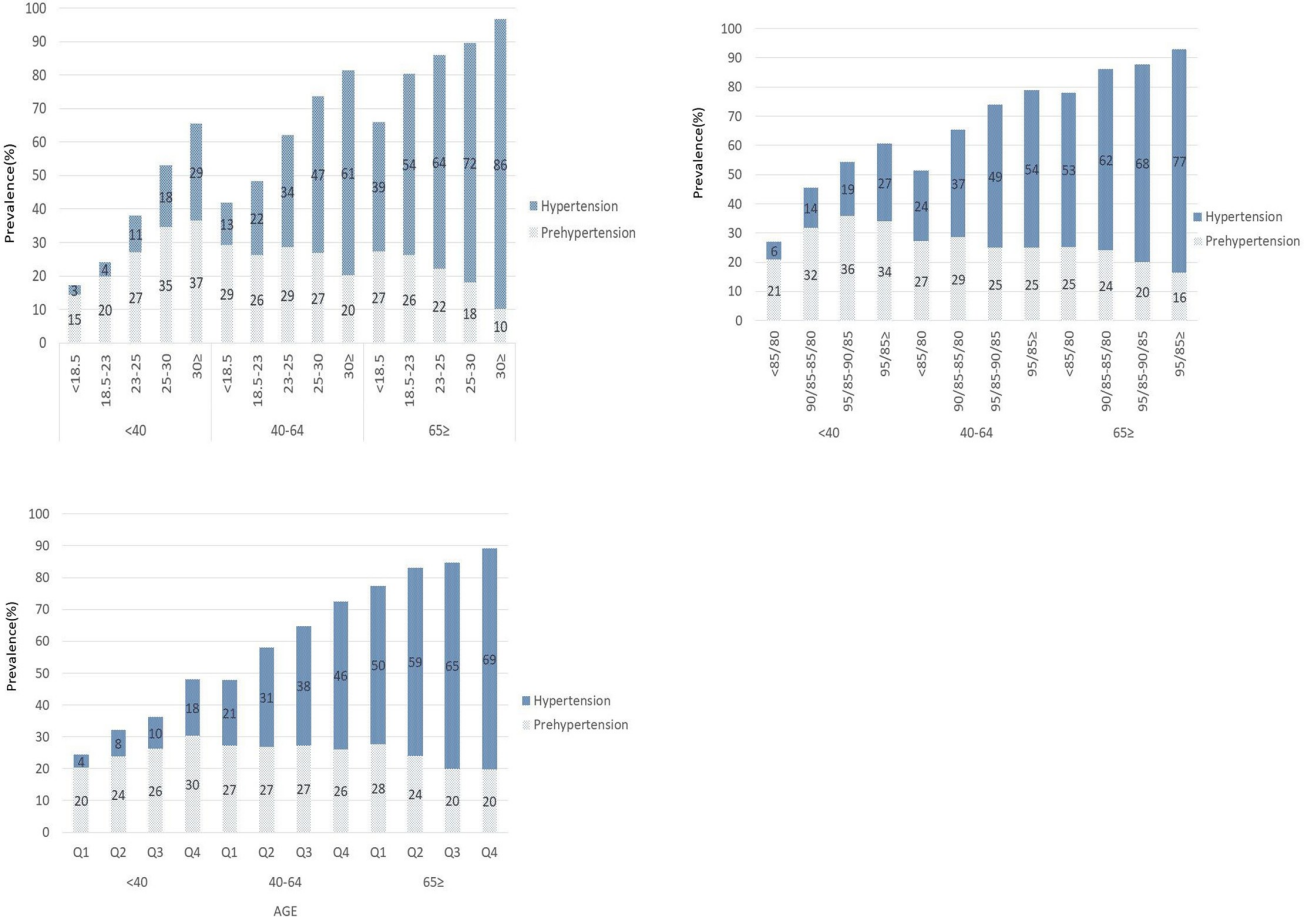
**a.** Prevalence of hypertension and prehypertension with increasing body mass index (BMI, kg/m^2^) categories by age group. **b.** Prevalence of hypertension and prehypertension with increasing waist circumference categories by age group. **c.** Prevalence of hypertension and prehypertension with increasing quartiles (Q1 through Q4) of percentage body fat by age group. Abbreviations: < 18.5; BMI less than 18.5, 18.5–23; BMI between 18.5 and 23, 25–30; BMI between 25 and 30, 30≥; BMI more than 30. Legend: Individuals with higher BMI, higher waist circumference, and higher PBF were more likely to have prehypertension in young adults less than 40 years of age, and to have hypertension in those 40 years of age or older (all *p* trend <0.001).

The prevalence of hypertension and prehypertension with respect to increasing BMI, WC, and PBF is shown in Fig 1A, 1B and 1C, respectively. Overall, the prevalence of hypertension increased as BMI, WC, and PBF increased (each *p trend* <0.001). This trend was also observed when stratified by age group (Fig 2A, 2B and 2C). Interestingly, the prevalence of prehypertension was also increased with increasing BMI, WC, and PBF in younger ages of less than 40 years (*p* trend <0.001). The difference of prevalence between hypertension and prehypertension was increased with increasing BMI, WC, and PBF in those in middle and old age (*p* trend <0.001).

The associations between various obesity parameters and prevalent hypertension are shown in Table 2. Hypertension was significantly associated with elevated BMI, WC, and PBF level after adjusting for potential confounders. Higher obesity parameters were associated with increased odds of hypertension (OR: 7.54; 95% CI, 5.89–9.65 for BMI ≥30 vs. 18.5–23, *p* trend <0.0001; OR: 3.97; 95% CI, 3.41–4.63 for WC ≥95 cm in males and ≥90 cm in females vs. <85 cm in males and <80 cm in females, *p* trend <0.0001; OR: 3.56; 95% CI, 3.05–4.15 for PBF, highest vs. lowest quartile, *p* trend <0.0001).

**Table 2:** Association of body mass index, waist circumference, and percentage body fat with prevalent hypertension.

	Hypertension			OR (95% CI)	
	No	Yes	*p*	Model1	Model2
**BMI (**kg/m^2^)			< .0001		
<18.5	6.2(0.3)	1.6(0.2)		0.46(0.32–0.67)	0.43(0.30–0.63)
18.5–23	46.5(0.6)	25.7(0.8)		1	1
23–25	22.3(0.5)	25(0.7)		1.77(1.56–2.00)	1.78(1.55–2.03)
25–30	22.2(0.5)	40.6(0.9)		3.06(2.71–3.47)	3.09(2.71–3.51)
≥30	2.8(0.2)	7(0.5)		6.17(4.87–7.83)	7.54(5.89–9.65)
*p for trend*				< .0001	< .0001
**WC (cm)**			< .0001		
<85 in male and <80 in female	66.1(0.6)	36.9(1.0)		1	1
85-<90 in male and 80-<85 in female	16.9(0.4)	22.2(0.7)		1.71(1.51–1.93)	1.75(1.54–1.99)
90-<95 in male and 85-<90 in female	9.7(0.3)	20.6(0.7)		2.66(2.31–3.07)	2.76(2.38–3.19)
95≥ in male and 90≥ in female	7.4(0.3)	20.3(0.8)		3.55(3.05–4.12)	3.97(3.41–4.63)
*p for trend*				< .0001	< .0001
**Percentage body fat**			< .0001		
Q1	30(0.8)	13.9(0.7)		1	1
Q2	25.9(0.5)	22.5(0.7)		1.86(1.59–2.16)	1.87(1.59–2.19)
Q3	23.3(0.5)	28.1(0.8)		2.37(2.04–2.76)	2.45(2.09–2.88)
Q4	20.7(0.8)	35.6(1.1)		3.44(2.95–4.01)	3.56(3.05–4.15)
*p for trend*				< .0001	< .0001

Data are presented as percentages (SE) or odds ratio (95% confidence interval).

Abbreviations: BMI, Body Mass Index; WC, Waist Circumference; Q, Quartile.

Model 1: Adjusted for age and sex.

Model 2: Adjusted for age, sex, smoking (never smoker, current smoker, past smoker), alcohol consumption (non-drinker, mild to moderate drinker, heavy drinker), physical activity (regular exercise, non-regular exercise, no exercise), living with spouse or not, income (quartiles), educational attainment (≤ 6 years, 7–12 years, ≥13 years), energy intake from fat, and sodium consumption.

When stratified by age group, the association between obesity parameters and hypertension was consistently stronger in the younger age group (< 40 years) (*p interaction =* 0.07 for BMI, *p interaction* = 0.004 for WC, *p interaction =* 0.10 for PBF level) (Table 3).

**Table 3:** Odds ratios and 95% confidence intervals for the association between obesity parameters and prevalent hypertension by age group.

	Age (year)			
	19–39 (n = 5301)	40–64 (n = 7491)	≥65 (n = 3571)	*P interaction*
**BMI(kg/m**^**2**^**)**				0.07
<18.5	1.00(0.42–2.40)	0.41(0.21–0.76)	0.40(0.24–0.66)	
18.5–23	1	1	1	
23–25	2.20(1.48–3.26)	1.57 (1.32–1.88)	1.50(1.16–1.93)	
25–30	3.77(2.52–5.64)	2.91 (2.45–3.46)	2.01(1.59–2.53)	
≥30	7.63(4.59–12.68)	7.27 (5.16–10.25)	4.99(2.39–10.42)	
P for trend	< .0001	< .0001	< .0001	
**WC (cm)**				0.004
<85 in male and <80 in female	1	1	1	
85-<90 in male and 80-<85 in female	2.04(1.43–2.93)	1.56(1.32–1.84)	1.41(1.12–1.78)	
90-<95 in male and 85-<90 in female	2.60(1.75–3.87)	2.80(2.31–3.41)	1.84(1.43–2.36)	
≥95 in male and ≥90 in female	4.80(3.30–6.98)	3.32(2.73–4.07)	2.54(1.92–3.35)	
P for trend	< .0001	< .0001	< .0001	
**Percentage body fat**				0.10
Q1	1	1	1	
Q2	1.88(1.22–2.89)	1.86(1.48–2.33)	1.68(1.28–2.19)	
Q3	2.47(1.58–3.85)	2.37(1.91–2.93)	2.07(1.59–2.68)	
Q4	4.28(2.93–6.26)	3.50(2.79–4.37)	2.21(1.69–2.91)	
P for trend	< .0001	< .0001	< .0001	

Abbreviations: BMI, body mass index; WC, waist circumference; Q, quartile

Adjusted for age, sex, smoking (never smoker, current smoker, past smoker), alcohol consumption (non-drinker, mild to moderate drinker, heavy drinker), physical activity (regular exercise, non-regular exercise, no exercise), living with spouse or not, income (quartiles), educational attainment (≤ 6 years, 7–12 years, ≥13 years), energy intake from fat, and sodium consumption.

Evidence related to the role of central obesity and body fat in the association between BMI and prevalent hypertension is shown in Table 4. Compared with those with normal BMI and WC, those with high BMI and high WC had the highest OR for prevalent hypertension (OR: 3.46; 95% CI, 3.07–3.90, *p interaction* = 0.06). Similar associations were found in the combination of BMI and PBF. Among individuals with normal BMI, the odds of hypertension was consistently elevated in individuals with the normal weight central obesity phenotype [normal BMI and high WC (WC≥90 in male, WC≥80 in female); OR: 1.89; 95% CI, 1.63–2.19]; and with normal weight obesity phenotype (normal BMI and highest quartile of BF; OR: 1.49; 95% CI, 1.25–1.77). Associations appear to be stronger in the younger age group (< 40 years), although there was no significant interaction.

**Table 4:** Combined associations of body mass index and other obesity parameters with prevalent hypertension.

	Hypertension		Age (year)				
	No	Yes	Model1	Model2	19–39	40–64	≥65
**Combination of BMI and WC**							
Normal BMI and WC	67.2(0.6)	39.5(0.9)	1	1	1	1	1
Normal BMI and elevated WC*	7.8(0.3)	12.9(0.6)	1.89(1.64,2.18)	1.89(1.63,2.19)	1.43(0.75–2.73)	1.70(1.39–2.08)	1.68(1.30–2.19)
Elevated BMI only†	8.1(0.3)	10.3(0.6)	2.28(1.93,2.71)	2.31(1.89,2.82)	2.22(1.44–3.44)	2.19(1.72–2.80)	1.59(0.93–2.70)
Both elevated BMI and WC	17(0.5)	37.3(0.9)	3.35(3.00,3.74)	3.46(3.07,3.90)	4.25(3.18–5.69)	3.16(2.72–3.68)	2.41(1.95–2.98)
*p interaction*			0.03	0.06	0.46	0.30	0.73
**Combination of BMI and PBF**							
Normal BMI and PBF	66.7(0.7)	42.6(1)	1	1	1	1	1
Normal BMI and elevated PBF‡	8.3(0.5)	9.8(0.6)	1.56(1.32,1.85)	1.49(1.25,1.77)	1.98(1.23–3.18)	1.49(1.19–1.86)	1.14(0.86–1.52)
Elevated BMI only†	12.6(0.4)	21.8(0.8)	2.55(2.25,2.88)	2.57(2.24,2.94)	2.75(1.83–4.13)	2.39(2.04–2.80)	1.94(1.46–2.56)
Both elevated BMI and PBF	12.5(0.4)	25.8(0.9)	3.32(2.94,3.75)	3.41(2.99,3.89)	4.42(3.24–6.04)	3.31(2.77–3.96)	2.02(1.60–2.54)
*p interaction*			0.96	0.11	0.35	0.51	0.65

Abbreviations: BMI, Body Mass Index; WC, waist circumference; PBF: percentage body fat

*Elevated WC: WC≥90 cm in male and WC≥80 cm in female

†Elevated BMI: BMI≥25 kg/m^2^

‡Elevated PBF: highest quartile of PBF

Data are presented as percentages (SE) or odds ratio (95% confidence interval).

Model 1: Adjusted for age and sex.

Model 2: Adjusted for age, sex, smoking (never smoker, current smoker, past smoker), alcohol consumption (non-drinker, mild to moderate drinker, heavy drinker), physical activity (regular exercise, non-regular exercise, no exercise), living with spouse or not, income (quartiles), educational attainment (≤ 6 years, 7–12 years, ≥13 years), energy intake from fat, and sodium consumption.

In the sensitivity analyses, these associations were consistent with using a new guideline (S1 and S2 Tables). No differential association was observed by diabetes, education, income, smoking habit, alcohol consumption, and exercise (S3, S4, and S5 Tables).

Sex-specific associations between obesity parameters and hypertension are shown in S6 to S8 Tables. Overall, there was no difference between sexes in the association of BMI, WC, and PBF with hypertension (S6 Table). However, associations differed by sex and age group (*p interaction =* 0.03 for BMI, *p interaction* = 0.06 for WC, *p interaction =* 0.003 for PBF level) and a stronger association between BMI and hypertension was clearly seen in women at younger ages of less than 40 years and older ages of 65 or more (S7 Table). The role of central obesity and body fat in the association between BMI and prevalent hypertension appeared to be stronger in women at younger ages of less than 40 years compared with men in the same age group (S8 Table).

## Discussion

In this nationally representative population-based study in South Korea, increasing BMI, WC, and PBF were significantly associated with an increased prevalence of hypertension, especially in young adults (< 40 years). Furthermore, we observed a positive association of high WC and PBF with hypertension in individuals within the normal range of BMI.

In addition to overall positive association of increased BMI, WC, and PBF with prevalent hypertension, we observed that higher abdominal obesity or body fat composition with normal BMI was associated with increased odds of hypertension. Moreover, the highest odds of hypertension were observed when the obese individuals also had abdominal obesity (Table 4). One epidemiologic study also found that young women had more prevalent hypertension if they had high WC [27]. The study also measured abdominal obesity using computed tomography (CT) scans to assess visceral fat, and it showed that central obesity-related visceral fat is a risk for hypertension [28]. These findings suggest that BMI alone has a limitation as a parameter to explain obesity-related hypertension. Recent studies have shown that WC, waist-to-hip ratio, and visceral fat measurement may be better discriminators of obesity-related complications than BMI [29,30,31]. It has been emphasized that central obesity has a stronger association with hypertension than general obesity. Increased abdominal fat representing central obesity is also known to be a more important factor in hypertension management [19,32].

Normal weight obesity represented by normal BMI with elevated PBF was suggested as another type of obesity that is related to increased cardiovascular risk in many studies. While individuals with higher body fat composition may be susceptible to metabolic disease, including hypertension [33], higher body fat in normal range of BMI was regarded to be a controversial risk factor for hypertension [34]. However, our study results showed that higher body fat was associated with increased odds of hypertension, suggesting that lowering body fat composition may increase the chance to control BP in normal-weight individuals [35]. Furthermore, the association between normal weight obesity and hypertension may differ by sex and age. In our stratified analysis by sex and age groups, the association was stronger in women than in men among young individuals (<40 years old), which is consistent with a prior study [36].

BMI was reported to lack the power to differentiate between body fat and lean body mass [37]. We also consider that body composition, such as muscle mass, could affect BP. Usually, muscle mass had an inverse relationship with the PBF [38]; therefore, individuals with a high PBF could have lower muscle mass, which may be related to high prevalence of hypertension.

In our study, people less than 40 years of age tended to have more prehypertension as BMI, WC, and PBF increased. Our findings suggest that younger age might require earlier control and management of obesity to prevent development of hypertension or delay hypertension once age increases. Young persons were found to have a stronger association between adiposity and hypertension [39]. Therefore, younger-aged subjects’ prehypertension with obesity may need to be screened and managed properly for preventing hypertension [40]. In addition, hypertension could be masked among prehypertensive obese young persons, and it might require regular BP checks for screening [41]. This population might have elevated BP at a certain time; therefore, elevated BP can be detected [42].

The limitations of our study include its cross-sectional design, and thus, no causal relationships could be obtained. To confirm the relationship between obesity status and hypertension management, long-term intervention studies are necessary. However, this study was based on a nationally representative sample of the Korean adult population, which is a major strength. In addition, data were collected based on standardized protocols to minimize the influence of measurement errors. Finally, we were able to assess potential effect modification by sex and age groups and to replicate the findings using a sensitivity analysis with a recently updated hypertension diagnosis guideline [43].

In conclusion, there was a positive association between various obesity parameters and hypertension in representative Korean adults. Even within the normal range of BMI, high WC and high PBF were associated with hypertension. Thus, measurement of BMI may not be sufficient to establish the association between obesity and hypertension. Our findings suggest that it is important to recognize and manage high-risk patients with proper obesity risk assessment [44] because hypertension in normal weight obesity could be easily neglected. Our study results also suggest that obese young people with prehypertension may require more attention and early appropriate management to control.

## Supporting information

S1 Table Association of body mass index, waist circumference, and percentage body fat with prevalent hypertension based on a new guideline^€^. (DOCX)

S2 Table Combined associations of body mass index and other obesity parameters with prevalent hypertension based on new guideline^€^. (DOCX)

S3 Table Subgroup analysis for the association between body mass index and prevalent hypertension. (DOCX)

S4 Table Subgroup analysis for the association between waist circumference and prevalent hypertension. (DOCX)

S5 Table Subgroup analysis for the association between percentage body fat and prevalent hypertension. (DOCX)

S6 Table Association of body mass index, waist circumference, and percentage body fat with prevalent hypertension by sex. (DOCX)

S7 Table Odds ratio and 95% confidence intervals for the association between obesity parameters and prevalent hypertension by sex and age group. (DOCX)

S8 Table Combined associations of body mass index and other obesity parameters with prevalent hypertension by sex and age group. (DOCX)
