# Supplementary material for: Obesity-related hypertension: Findings from The Korea National Health and Nutrition Examination Survey 2008–2010
Source: PLoS One. 2020 Apr 21;15(4):e0230616. doi: 10.1371/journal.pone.0230616 (PMC7173931; doi:10.1371/journal.pone.0230616)
Supplement: S1 Table — (DOCX) [file pone.0230616.s001.docx]

Supplemental Table 1. Association of body mass index, waist circumference, and percentage body fat with prevalent hypertension based on a new guideline^€^

|  | Hypertension | | | | OR (95% CI) | |
| --- | --- | --- | --- | --- | --- | --- |
|  | No | Yes | p | Model1 | | Model2 |
| **BMI (**kg/m^2^) |  |  | <.0001 |  | |  |
| <18.5 | 7.2(0.4) | 2.5(0.2) |  | 0.72(0.56-0.92) | | 0.71(0.54-0.92) |
| 18.5-23 | 50.7(0.7) | 30.1(0.7) |  | 1 | | 1 |
| 23-25 | 21.2(0.5) | 25(0.6) |  | 1.66(1.49-1.85) | | 1.61(1.43-1.80) |
| 25-30 | 18.7(0.6) | 36.5(0.6) |  | 2.68(2.41-2.98) | | 2.82(2.50-3.17) |
| ≥30 | 2.2(0.2) | 5.9(0.3) |  | 5.44(4.29-6.90) | | 6.06(4.69-7.82) |
| *p for trend* |  |  |  | <.0001 | | <.0001 |
| **WC (cm)** |  |  | <.0001 |  | |  |
| <85 in male and <80 in female | 70.6(0.7) | 44.6(1) |  | 1 | | 1 |
| 85-<90 in male and 80-<85 in female | 15.2(0.5) | 21.7(0.6) |  | 1.67(1.50-1.86) | | 1.69(1.50-1.90) |
| 90-<95 in male and 85-<90 in female | 8.3(0.4) | 17.3(0.5) |  | 2.27(1.98-2.61) | | 2.34(2.01-2.73) |
| ≥95 in male and ≥90 in female | 5.9(0.3) | 16.4(0.6) |  | 3.35(2.88-3.89) | | 3.56(3.04-4.18) |
| *p for trend* |  |  |  | <.0001 | | <.0001 |
| **Percentage body fat** |  |  | <.0001 |  | |  |
| Q1 | 32.8(0.9) | 17.8(0.7) |  | 1 | | 1 |
| Q2 | 26.2(0.6) | 23.7(0.6) |  | 1.55(1.37-1.74) | | 1.62(1.42-1.84) |
| Q3 | 22.4(0.6) | 27(0.6) |  | 1.93(1.70-2.18) | | 2.04(1.79-2.34) |
| Q4 | 18.6(0.8) | 31.5(1) |  | 2.8(2.47-3.18) | | 2.90(2.54-3.32) |
| *p for trend* |  |  |  | <.0001 | | <.0001 |

Data are presented as percentages (SE) or odds ratio (95% confidence interval).

Abbreviations: BMI, Body Mass Index; WC, Waist Circumference; Q, Quartile.

Model 1: Adjusted for age and sex.

Model 2: Adjusted for age, sex, smoking (never smoker, current smoker, past smoker), alcohol consumption (non-drinker, mild to moderate drinker, heavy drinker), physical activity (regular exercise, non-regular exercise, no exercise), living with spouse or not, income (quartiles), educational attainment (≤ 6 years, 7-12 years, ≥13 years), energy intake from fat, and sodium consumption.

^€^2017 new guideline set to lower the definition of hypertension as systolic blood pressure≥130 mmHg or diastolic blood pressure≥80 mmHg.
