# Supplementary material for: Obesity-related hypertension: Findings from The Korea National Health and Nutrition Examination Survey 2008–2010
Source: PLoS One. 2020 Apr 21;15(4):e0230616. doi: 10.1371/journal.pone.0230616 (PMC7173931; doi:10.1371/journal.pone.0230616)
Supplement: S2 Table — (DOCX) [file pone.0230616.s002.docx]

Supplemental Table 2. Combined associations of body mass index and other obesity parameters with prevalent hypertension based on new guideline^€^

|  | Hypertension | | Age (year) | | | | |
| --- | --- | --- | --- | --- | --- | --- | --- |
|  | No | Yes | Model1 | Model2 | 19-39 | 40-64 | ≥65 |
| **Combination of BMI and WC** |  |  |  |  |  |  |  |
| Normal BMI and WC | 71.4(0.7) | 46.8(0.8) | 1 | 1 | 1 | 1 | 1 |
| Elevated WC only* | 7.7(0.3) | 10.8(0.5) | 1.71(1.49-1.97) | 1.64(1.41-1.91) | 1.17(0.77-1.79) | 1.45(1.19-1.78) | 1.83(1.39-2.40) |
| Elevated BMI only† | 6.5(0.4) | 11(0.5) | 2.17(1.85-2.56) | 2.37(1.98-2.85) | 2.13(1.59-2.86) | 2.43(1.89-3.13) | 1.68(0.85-3.30) |
| Both elevated BMI and WC | 14.4(0.5) | 31.4(0.8) | 2.90(2.59-3.24) | 3.05(2.70-3.45) | 3.05(2.42-3.84) | 2.91(2.50-3.39) | 2.31(1.79-3.00) |
| *p interaction* |  |  | 0.02 | 0.04 | 0.45 | 0.23 | 0.45 |
|  |  |  |  |  |  |  |  |
| **Combination of BMI and PBF** | 70.7(0.8) | 48.6(0.8) | 1 | 1 | 1 | 1 | 1 |
| Normal BMI and PBF | 8.4(0.6) | 9(0.5) | 1.37(1.17-1.60) | 1.24(1.05-1.46) | 0.99(0.72-1.38) | 1.41(1.13-1.76) | 1.08(0.80-1.46) |
| PBF highest quartile | 10.7(0.5) | 19.9(0.6) | 2.20(1.95-2.50) | 2.29(1.99-2.63) | 2.05(1.55-2.72) | 2.34(1.97-2.78) | 1.55(1.11-2.16) |
| Elevated BMI* | 10.2(0.5) | 22.5(0.7) | 3.02(2.69-3.40) | 3.24(2.86-3.68) | 3.19(2.58-3.95) | 3.32(2.79-3.96) | 2.14(1.61-2.83) |
| *p interaction* | |  | 0.97 | 0.26 | 0.05 | 0.97 | 0.34 |
|  |  |  |  |  |  |  |  |

Abbreviations: BMI, Body Mass Index; WC, waist circumference; PBF: percentage body fat

*Elevated WC: WC≥90 cm in male and WC≥80 cm in female

†Elevated BMI: BMI≥25 kg/m^2^

Data are presented as percentages (SE) or odds ratio (95% confidence interval).

Model 1: Adjusted for age and sex.

Model 2: Adjusted for age, sex, smoking (never smoker, current smoker, past smoker), alcohol consumption (non-drinker, mild to moderate drinker, heavy drinker), physical activity (regular exercise, non-regular exercise, no exercise), living with spouse or not, income (quartiles), educational attainment (≤ 6 years, 7-12 years, ≥13 years), energy intake from fat, and sodium consumption.

^€^2017 new guideline set to lower the definition of hypertension as systolic blood pressure≥130 mmHg or diastolic blood pressure≥80 mmHg.
