# Supplementary material for: Obesity-related hypertension: Findings from The Korea National Health and Nutrition Examination Survey 2008–2010
Source: PLoS One. 2020 Apr 21;15(4):e0230616. doi: 10.1371/journal.pone.0230616 (PMC7173931; doi:10.1371/journal.pone.0230616)
Supplement: S4 Table — (DOCX) [file pone.0230616.s004.docx]

Supplemental Table 4. Subgroup analysis for the association between waist circumference and prevalent hypertension

|  |  | **Waist circumference** | | | |  | |
| --- | --- | --- | --- | --- | --- | --- | --- |
| Characteristic | | <85 in male and <80 in female | 85-<90 in male and 80-<85 in female | 90-<95 in male and 85-<90 in female | ≥95 in male and ≥90 in female | | *P interaction* |
| **Diabetes mellitus** | No | 1 | 1.62(1.41-1.87) | 2.72(2.31-3.20) | 3.56(2.94-4.31) | | 0.53 |
|  | Yes | 1 | 1.94(1.31-2.85) | 2.50(1.66-3.76) | 2.89(1.95-4.28) | |  |
| **Education (year)** | <9 | 1 | 1.501.25-1.79) | 2.34(1.93-2.82) | 2.83(2.29-3.51) | | 0.27 |
|  | ≥10 | 1 | 1.72(1.42-2.07) | 2.76(2.23-3.42) | 4.05(3.23-5.09) | |  |
| **Income** | Others | 1 | 1.70(1.46-1.96) | 2.77(2.33-3.29) | 3.73(3.12-4.47) | | 0.87 |
|  | Lowest | 1 | 1.77(1.34-2.33) | 2.55(1.93-3.37) | 3.35(2.45-4.58) | |  |
| **Smoking** | Never or Past | 1 | 1.72(1.49-1.99) | 2.84(2.40-3.36) | 3.73(3.12-4.48) | | 0.88 |
|  | Current | 1 | 1.60(1.18-2.17) | 2.41(1.74-3.34) | 3.42(2.40-4.88) | |  |
| **Alcohol consumption** | Non-to moderate drinker | 1 | 1.78(1.55-2.05) | 2.92(2.48-3.44) | 3.82(3.22-4.52) | | 0.22 |
|  | Heavy drinker | 1 | 1.21(0.79-1.84) | 1.65(1.04-2.63) | 2.77(1.64-4.65) | |  |
| **Regular physical activity** | No | 1 | 1.78(1.52-2.09) | 2.72(2.29-3.24) | 3.57(2.95-4.32) | | 0.56 |
|  | Yes | 1 | 1.56(1.22-2.01) | 2.91(2.19-3.88) | 4.18(3.00-5.82) | |  |

Data are presented as odds ratio (95% confidence interval).

Adjusted for age, sex, smoking (never smoker, current smoker, past smoker), alcohol consumption (non-drinker, mild to moderate drinker, heavy drinker), physical activity (regular exercise, non-regular exercise, no exercise), living with spouse or not, income (quartiles), educational attainment (≤ 6 years, 7-12 years, ≥13 years), energy intake from fat, and sodium consumption ), except a stratifying variable.
