# Supplementary material for: Obesity-related hypertension: Findings from The Korea National Health and Nutrition Examination Survey 2008–2010
Source: PLoS One. 2020 Apr 21;15(4):e0230616. doi: 10.1371/journal.pone.0230616 (PMC7173931; doi:10.1371/journal.pone.0230616)
Supplement: S5 Table — (DOCX) [file pone.0230616.s005.docx]

Supplemental Table 5. Subgroup analysis for the association between percentage body fat and prevalent hypertension

|  |  | **Percentage body fat** (quartiles) | | | |  | |
| --- | --- | --- | --- | --- | --- | --- | --- |
| Characteristic | | Q1 | Q2 | Q3 | Q4 | | *P interaction* |
| **Diabetes mellitus** | No | 1 | 1.97(1.66-2.34) | 2.50(2.11-2.95) | 3.68(3.11-4.34) | | 0.37 |
|  | Yes | 1 | 1.32(0.83-2.09) | 1.85(1.12-3.07) | 2.37(1.47-3.84) | |  |
| **Education (year)** | <9 | 1 | 1.54(1.21-1.96) | 2.01(1.59-2.54) | 2.66(2.12-3.33) | | 0.45 |
|  | ≥10 | 1 | 2.18(1.72-2.77) | 2.71(2.15-3.41) | 4.06(3.22-5.13) | |  |
| **Income** | Others | 1 | 1.95(1.60-2.37) | 2.54(2.10-3.07) | 3.69(3.06-4.44) | | 0.92 |
|  | Lowest | 1 | 1.83(1.32-2.53) | 2.33(1.68-3.21) | 3.01(2.19-4.09) | |  |
| **Smoking** | Never or Past | 1 | 1.87(1.56-2.28) | 2.35(1.97-2.80) | 3.31(2.76-3.97) | | 0.47 |
|  | Current | 1 | 1.96(1.42-2.70) | 2.69(1.90-3.80) | 3.86(2.80-5.33) | |  |
| **Alcohol consumption** | Non-to moderate drinker | 1 | 1.82(1.52-2.18) | 2.42(2.04-2.89) | 3.38(2.86-4.00) | | 0.22 |
|  | Heavy drinker | 1 | 2.76(1.70-4.48) | 2.76(1.68-4.52) | 5.08(3.06-8.45) | |  |
| **Regular physical activity** | No | 1 | 1.96(1.60-2.39) | 2.38(1.96-2.90) | 3.46(2.85-4.22) | | 0.61 |
|  | Yes | 1 | 1.85(1.38-2.48) | 2.81(2.09-3.79) | 3.92(2.88-5.32) | |  |

Data are presented as odds ratio (95% confidence interval).

Adjusted for age, sex, smoking (never smoker, current smoker, past smoker), alcohol consumption (non-drinker, mild to moderate drinker, heavy drinker), physical activity (regular exercise, non-regular exercise, no exercise), living with spouse or not, income (quartiles), educational attainment (≤ 6 years, 7-12 years, ≥13 years), energy intake from fat, and sodium consumption ), except a stratifying variable.
