# Supplementary material for: Obesity-related hypertension: Findings from The Korea National Health and Nutrition Examination Survey 2008–2010
Source: PLoS One. 2020 Apr 21;15(4):e0230616. doi: 10.1371/journal.pone.0230616 (PMC7173931; doi:10.1371/journal.pone.0230616)
Supplement: S6 Table — (DOCX) [file pone.0230616.s006.docx]

Supplemental Table 6. Association of body mass index, waist circumference, and percentage body fat with prevalent hypertension by sex

|  |  |  | | | Hypertension | |  | OR (95% CI) | |
| --- | --- | --- | --- | --- | --- | --- | --- | --- | --- |
|  |  |  | | | No | Yes | *p* | Model1 | Model2 |
| **BMI (**kg/m^2^) | **Male** | | <18.5 | 3.8(0.3) | | 1.8(0.3) | <.0001 | 0.63(0.40,0.99) | 0.592(0.35,0.99) |
|  |  | | 18.5-23 | 40.7(0.9) | | 24.5(1.1) |  | 1 | 1 |
|  |  | | 23-25 | 24.9(0.7) | | 25.5(1) |  | 1.78(1.505,2.1) | 1.74(1.44,2.09) |
|  |  | | 25-30 | 27.9(0.8) | | 41.9(1.3) |  | 2.84(2.40,3.36) | 2.89(2.38,3.50) |
|  |  | | ≥30 | 2.7(0.3) | | 6.2(0.6) |  | 6.66(4.79,9.25) | 7.59(5.19,11.08) |
|  |  | | *p for trend* |  | |  |  | <.0001 | <.0001 |
|  | **Female** | | <18.5 | 8.2(0.4) | | 1.4(0.3) | <.0001 | 0.39(0.25,0.62) | 0.33(0.19,0.54) |
|  |  | | 18.5-23 | 51.6(0.8) | | 27.3(1) |  | 1 | 1 |
|  |  | | 23-25 | 20.1(0.6) | | 24.4(1) |  | 1.70(1.43,2.02) | 1.61(1.34,1.92) |
|  |  | | 25-30 | 17.1(0.6) | | 38.8(1.2) |  | 3.03(2.58,3.57) | 2.79(2.36,3.31) |
|  |  | | ≥30 | 3(0.3) | | 8.1(0.7) |  | 6.42(4.72,8.75) | 7.15(5.14,9.96) |
|  |  | | *p for trend* |  | |  |  | <.0001 | <.0001 |
| **WC (cm)** | **Male** | | <85 | 61.8(0.9) | | 40.2(1.3) | <.0001 | 1 | 1 |
|  |  | | 85-<90 | 19.5(0.7) | | 23(1) |  | 1.59(1.38,1.86) | 1.62(1.35,1.94) |
|  |  | | 90-<95 | 11.2(0.5) | | 20.4(1) |  | 2.36(1.97,2.82) | 2.45(2.00,2.99) |
|  |  | | 95≥ | 7.5(0.4) | | 16.4(0.9) |  | 3.29(2.72,3.99) | 3.53(2.81,4.42) |
|  |  | | *p for trend* |  | |  |  | <.0001 | <.0001 |
|  | **Female** | | <80 | 69.9(0.8) | | 32.7(1.2) | <.0001 | 1 | 1 |
|  |  | | 80-<85 | 14.5(0.5) | | 21.2(1) |  | 1.81(1.51,2.16) | 1.68(1.40,2.02) |
|  |  | | 85-<90 | 8.3(0.4) | | 20.7(0.9) |  | 2.89(2.39,3.48) | 2.74(2.25,3.34) |
|  |  | | 90≥ | 7.3(0.4) | | 25.3(1.2) |  | 3.83(3.16,4.63) | 3.90(3.19,4.78) |
|  |  | | *p for trend* |  | |  |  | <.0001 | <.0001 |
| **Percentage body fat** | **Male** | | Q1 | 30.3(1) | | 14.5(0.9) | <.0001 | 1 | 1 |
|  |  | | Q2 | 25.2(0.8) | | 24.4(1) |  | 1.89(1.57,2.28) | 1.94(1.56,2.39) |
|  |  | | Q3 | 23.6(0.8) | | 27.4(1.1) |  | 2.22(1.83,2.69) | 2.25(1.81,2.81) |
|  |  | | Q4 | 20.9(0.9) | | 33.6(1.4) |  | 3.40(2.84,4.06) | 3.66(2.98,4.49) |
|  |  | | *p for trend* |  | |  |  | <.0001 | <.0001 |
|  | **Female** | | Q1 | 29.8(0.9) | | 13(0.9) | <.0001 | 1 | 1 |
|  |  | | Q2 | 26.5(0.7) | | 20.2(0.9) |  | 1.69(1.36,2.10) | 1.66(1.31,2.11) |
|  |  | Q3 | | | 23(0.7) | 28.8(1.1) |  | 2.42(1.99,2.93) | 2.40(1.95,2.96) |
|  |  | Q4 | | | 20.6(0.8) | 38(1.3) |  | 3.15(2.55,3.89) | 2.91(2.32,3.66) |
|  |  | *p for trend* | | |  |  |  | <.0001 | <.0001 |

Data are presented as percentages (SE) or odds ratio (95% confidence interval).

Abbreviations: BMI, Body Mass Index; WC, Waist Circumference; Q, Quartile.

Model 1: Adjusted for age.

Model 2: Adjusted for age, smoking (never smoker, current smoker, past smoker), alcohol consumption (non-drinker, mild to moderate drinker, heavy drinker), physical activity (regular exercise, non-regular exercise, no exercise), living with spouse or not, income (quartiles), educational attainment (≤ 6 years, 7-12 years, ≥13 years), energy intake from fat, and sodium consumption.
