# Supplementary material for: Obesity-related hypertension: Findings from The Korea National Health and Nutrition Examination Survey 2008–2010
Source: PLoS One. 2020 Apr 21;15(4):e0230616. doi: 10.1371/journal.pone.0230616 (PMC7173931; doi:10.1371/journal.pone.0230616)
Supplement: S7 Table — (DOCX) [file pone.0230616.s007.docx]

Supplemental Table 7.  Odds ratio and 95% confidence intervals for the association between obesity parameters and prevalent hypertension by sex and age group

|  |  |  | No | Yes | OR (95% CI) | *P interaction* |
| --- | --- | --- | --- | --- | --- | --- |
| **BMI(kg/m^2^)** | Male, Age 19-39 | <18.5 | 3.9(0.5) | 2.1(0.9) | 1.22(0.40,3.75) | 0.03 |
|  |  | 18.5-23 | 41.9(1.2) | 20.4(2.7) | 1 |  |
|  |  | 23-25 | 23.2(1) | 23(2.4) | 2.12(1.38,3.26) |  |
|  |  | 25-30 | 27.1(1.1) | 41.1(2.9) | 3.25(2.08,5.00) |  |
|  |  | 30- | 3.9(0.5) | 13.4(1.9) | 8.44(4.72,15.1) |  |
|  |  | *p for trend* |  |  | <.0001 |  |
|  | Male, Age 40-64 | <18.5 | 2.7(0.4) | 1.2(0.3) | 0.86(0.42,1.76) |  |
|  |  | 18.5-23 | 37.6(1.3) | 20.6(1.3) | 1 |  |
|  |  | 23-25 | 27.6(1.2) | 25.7(1.3) | 1.69(1.30,2.20) |  |
|  |  | 25-30 | 30.8(1.2) | 47.4(1.6) | 2.99(2.34,3.83) |  |
|  |  | 30- | 1.3(0.3) | 5.1(0.7) | 10.26(5.41,19.47) |  |
|  |  | *p for trend* |  |  | <.0001 |  |
|  | Male, Age 65≥ | <18.5 | 9.7(1.3) | 3.4(0.8) | 0.32(0.16,0.62) |  |
|  |  | 18.5-23 | 48.7(2.3) | 40.8(2.3) | 1 |  |
|  |  | 23-25 | 22.7(1.9) | 27.5(2) | 1.41(0.99,1.99) |  |
|  |  | 25-30 | 18.2(1.6) | 26.5(2) | 1.72(1.23,2.42) |  |
|  |  | 30- | 0.7(0.5) | 1.9(0.5) | 3.37(0.91,12.45) |  |
|  |  | *p for trend* |  |  | <.0001 |  |
|  | Female,Age 19-39 | <18.5 | 13(0.8) | 3.3(2) | 0.83(0.22,3.11) |  |
|  |  | 18.5-23 | 58(1) | 24.5(4.7) | 1 |  |
|  |  | 23-25 | 14.5(0.7) | 13.9(4) | 2.05(0.95,4.42) |  |
|  |  | 25-30 | 11.2(0.7) | 40.3(5.8) | 6.44(3.33,12.44) |  |
|  |  | 30- | 3.3(0.4) | 18(5.1) | 12.75(5.48,29.68) |  |
|  |  | *p for trend* |  |  | <.0001 |  |
|  | Female,Age 40-64 | <18.5 | 3.3(0.4) | 0.3(0.1) | 0.13(0.04,0.41) |  |
|  |  | 18.5-23 | 45.5(1.2) | 26.2(1.5) | 1 |  |
|  |  | 23-25 | 25.8(0.9) | 25.6(1.5) | 1.41(1.11,1.78) |  |
|  |  | 25-30 | 22.5(0.9) | 39.3(1.6) | 2.49(1.99,3.10) |  |
|  |  | 30- | 2.9(0.4) | 8.6(1) | 6.63(4.34,10.15) |  |
|  |  | *p for trend* |  |  | <.0001 |  |
|  | Female, Age 65≥ | <18.5 | 5.4(1) | 2.6(0.5) | 0.49(0.25,0.95) |  |
|  |  | 18.5-23 | 43.6(2.2) | 28.9(1.5) | 1 |  |
|  |  | 23-25 | 24.2(1.9) | 24.2(1.3) | 1.66(1.22,2.27) |  |
|  |  | 25-30 | 25.2(1.8) | 38(1.7) | 2.25(1.69,2.99) |  |
|  |  | 30- | 1.7(0.6) | 6.4(0.9) | 6.52(2.78,15.3) |  |
|  |  | *p for trend* |  |  | <.0001 |  |
| **WC (cm)** | Male, Age 19-39 | <85 | 67.1(1.1) | 44.9(3) | 1 | 0.06 |
|  |  | 85-<90 | 16.4(0.9) | 20.2(2.4) | 1.99(1.34,2.94) |  |
|  |  | 90-<95 | 9.1(0.7) | 14.1(1.9) | 2.47(1.60,3.79) |  |
|  |  | 95≥ | 7.5(0.7) | 20.8(2.3) | 4.74(3.18,7.63) |  |
|  |  | *p for trend* |  |  | <.0001 |  |
|  | Male, Age 40-64 | <85 | 55.6(1.3) | 36.7(1.7) | 1 |  |
|  |  | 85-<90 | 23.5(1.1) | 24.6(1.3) | 1.51(1.21,1.89) |  |
|  |  | 90-<95 | 13.4(0.8) | 23.2(1.4) | 2.62(2.014,3.41) |  |
|  |  | 95≥ | 7.6(0.7) | 15.6(1.1) | 3.09(2.28,4.17) |  |
|  |  | *p for trend* |  |  | <.0001 |  |
|  | Male, Age 65≥ | <85 | 57.3(2.4) | 45.9(2.3) | 1 |  |
|  |  | 85-<90 | 20.7(2) | 21.1(1.7) | 1.22(0.86,1.72) |  |
|  |  | 90-<95 | 15.3(1.6) | 18.9(1.6) | 1.55(1.11,2.17) |  |
|  |  | 95≥ | 6.7(1.3) | 14.1(1.4) | 2.78(1.69,4.55) |  |
|  |  | *p for trend* |  |  | <.0001 |  |
|  | Female,Age 19-39 | <80 | 81.2(0.9) | 43.9(5.8) | 1 |  |
|  |  | 80-<85 | 9.3(0.6) | 13.8(3.7) | 2.48(1.32,4.67) |  |
|  |  | 85-<90 | 4.5(0.4) | 17.9(5) | 4.66(2.08,10.45) |  |
|  |  | 90≥ | 5(0.4) | 24.4(5.5) | 8.29(4.03,17.05) |  |
|  |  | *p for trend* |  |  | <.0001 |  |
|  | Female,Age 40-64 | <80 | 60.6(1.1) | 34.4(1.6) | 1 |  |
|  |  | 80-<85 | 19.5(0.9) | 22.2(1.5) | 1.47(1.15,1.87) |  |
|  |  | 85-<90 | 11.3(0.7) | 21.3(1.4) | 2.51(1.95,3.22) |  |
|  |  | 90≥ | 8.6(0.6) | 22.1(1.6) | 3.68(2.88,4.69) |  |
|  |  | *p for trend* |  |  | <.0001 |  |
|  | Female,Age 65≥ | <80 | 46.6(2.3) | 29.4(1.6) | 1 |  |
|  |  | 80-<85 | 21.3(1.8) | 20.9(1.3) | 1.71(1.25,2.35) |  |
|  |  | 85-<90 | 15.6(1.6) | 20.3(1.3) | 2.2(1.63,3.08) |  |
|  |  | 90≥ | 16.5(1.7) | 29.4(1.5) | 2.92(2.12,4.00) |  |
|  |  | *p for trend* |  |  | <.0001 |  |
| **Percentage body fat** | Male, Age 19-39 | Q1 | 32.5(1.3) | 13.5(2.3) | 1 | 0.003 |
|  |  | Q2 | 22.5(1) | 22(2.4) | 2.35(1.43,3.84) |  |
|  |  | Q3 | 22.1(1.1) | 22.4(2.5) | 2.72(1.60,4.61) |  |
|  |  | Q4 | 22.9(1.2) | 42.1(3.1) | 4.82(3.06,7.59) |  |
|  |  | *p for trend* |  |  | <.0001 |  |
|  | Male, Age 40-64 | Q1 | 27.6(1.5) | 13.6(1.1) | 1 |  |
|  |  | Q2 | 29(1.2) | 26.5(1.4) | 1.96(1.48,2.60) |  |
|  |  | Q3 | 25.2(1.2) | 29.4(1.5) | 2.26(1.68,3.04) |  |
|  |  | Q4 | 18.1(1.2) | 30.5(1.7) | 3.74(2.77,5.05) |  |
|  |  | *p for trend* |  |  | <.0001 |  |
|  | Male, Age 65≥ | Q1 | 28(2.3) | 18.5(1.9) | 1 |  |
|  |  | Q2 | 24.3(2) | 20.5(1.6) | 1.22(0.81,1.84) |  |
|  |  | Q3 | 26.2(2) | 27.1(1.8) | 1.52(1.051,2.2) |  |
|  |  | Q4 | 21.5(1.9) | 34(2.1) | 2.35(1.6,3.45) |  |
|  |  | *p for trend* |  |  | `<.0001 |  |
|  | Female,Age 19-39 | Q1 | 35.8(1.2) | 17.6(4.2) | 1 |  |
|  |  | Q2 | 26.2(0.9) | 14.6(4.1) | 0.99(0.45,2.20) |  |
|  |  | Q3 | 19.5(0.9) | 22.1(4.8) | 2.06(1.4,4.08) |  |
|  |  | Q4 | 18.4(1.1) | 45.6(6.2) | 4.83(2.39,9.77) |  |
|  |  | *p for trend* |  |  | <.0001 |  |
|  | Female,Age 40-64 | Q1 | 23.6(1) | 11(1) | 1 |  |
|  |  | Q2 | 27.9(1) | 21.7(1.4) | 1.447(1.07,1.96) |  |
|  |  | Q3 | 27.3(0.9) | 31.5(1.6) | 2.02(1.56,2.62) |  |
|  |  | Q4 | 21.2(1.1) | 35.8(1.8) | 2.67(2.05,3.49) |  |
|  |  | *p for trend* |  |  | <.0001 |  |
|  | Female,Age 65≥ | Q1 | 25.8(2.3) | 14.8(1.3) | 1 |  |
|  |  | Q2 | 20.6(1.9) | 19(1.2) | 2.04(1.38,2.99) |  |
|  |  | Q3 | 21.5(1.7) | 26.4(1.5) | 2.67(1.88,3.79) |  |
|  |  | Q4 | 32.2(2.4) | 39.8(1.8) | 2.19(1.52,3.19) |  |
|  |  | *p for trend* |  |  | <.0001 |  |

Abbreviations: BMI, body mass index; WC, waist circumference; Q, quartile

Adjusted for age, sex, smoking (never smoker, current smoker, past smoker), alcohol consumption (non-drinker, mild to moderate drinker, heavy drinker), physical activity (regular exercise, non-regular exercise, no exercise), living with spouse or not, income (quartiles), educational attainment (≤ 6 years, 7-12 years, ≥13 years), energy intake from fat, and sodium consumption.
