# Supplementary material for: Obesity-related hypertension: Findings from The Korea National Health and Nutrition Examination Survey 2008–2010
Source: PLoS One. 2020 Apr 21;15(4):e0230616. doi: 10.1371/journal.pone.0230616 (PMC7173931; doi:10.1371/journal.pone.0230616)
Supplement: S8 Table — (DOCX) [file pone.0230616.s008.docx]

Supplement Table 8. Combined associations of body mass index and other obesity parameters with prevalent hypertension by sex and age group

|  |  | Hypertension | |  | | Age (year) | | |
| --- | --- | --- | --- | --- | --- | --- | --- | --- |
|  |  | No | Yes | Model1 | Model2 | 19-39 | 40-64 | ≥65 |
|  | **Combination of BMI and WC** |  |  |  |  |  |  |  |
| Male | Normal BMI and WC | 67.2(0.6) | 39.5(0.9) | 1 | 1 | 1 | 1 | 1 |
|  | Elevated WC only* | 7.8(0.3) | 12.9(0.6) | 1.76(1.32,2.33) | 1.89(1.63,2.19) | 1.86(0.64,5.38) | 1.70(1.13,2.58) | 1.73(1.14,2.63) |
|  | Elevated BMI only† | 8.1(0.3) | 10.3(0.6) | 2.02(1.69,2.43) | 2.31(1.89,2.82) | 2.02(1.28,3.17) | 2.05(1.57,2.67) | 1.63(0.90,2.92) |
|  | Both elevated BMI and WC | 17(0.5) | 37.3(0.9) | 2.93(2.51,3.43) | 3.46(3.07,3.90) | 3.54(2.52,4.97) | 3.12(2.49,3.89) | 1.92(1.38,2.66) |
|  | *p interaction* |  |  | 0.25 | 0.25 | 0.92 | 0.66 | 0.34 |
| Female | Normal BMI and WC | 67.3(0.8) | 30.2(1.2) | 1 | 1 | 1 | 1 | 1 |
|  | Elevated WC only* | 12.6(0.5) | 23(1.1) | 1.80(1.51,2.2) | 1.77(1.39,2.25) | 1.49(0.61,3.63) | 1.52(1.21,1.92) | 1.79(1.31,2.44) |
|  | Elevated BMI only† | 2.5(0.2) | 2.5(0.4) | 2.56(1.70,3.84) | 2.31(1.90,2.80) | 4.73(1.84,12.21) | 2.19(1.41,3.41) | 1.44(0.48,4.30) |
|  | Both elevated BMI and WC | 17.6(0.6) | 44.3(1.2) | 3.54(3.05,4.11) | 3.35(2.75,4.09) | 7.21(4.05,12.82) | 2.98(2.34,3.64) | 2.80(2.12,3.68) |
|  | *p interaction* |  |  | 0.42 | 0.48 | 0.97 | 0.66 | 0.90 |
|  | **Combination of BMI and PBF** |  |  |  |  |  |  |  |
| Male | Normal BMI and PBF | 70.1(2.2) | 55.9(2.3) | 1 | 1 | 1 | 1 | 1 |
|  | PBF highest quartile | 11(1.4) | 15.7(1.6) | 1.77(1.41,2.24) | 1.77(1.39,2.25) | 1.92(1.09,3.38) | 1.77(1.244,2.51) | 1.75(1.17,2.61) |
|  | Elevated BMI* | 8.4(1.3) | 10.1(1.2) | 2.30(1.95,2.72) | 2.31(1.90,2.80) | 2.27(1.45,3.55) | 2.32(1.853,2.91) | 1.49(0.95,2.35) |
|  | Both elevated BMI and PBF | 10.5(1.3) | 18.2(1.7) | 3.10(2.62,3.67) | 3.53(2.75,4.09) | 3.70(2.59,5.27) | 3.55(2.707,4.67) | 2.24(1.56,3.22) |
|  | *p interaction* |  |  | 0.08 | 0.12 | 0.65 | 0.55 | 0.66 |
|  | **Combination of BMI and PBF** |  |  |  |  |  |  |  |
| Female | Normal BMI and PBF | 70.5(0.8) | 42.9(1.2) | 1 | 1 | 1 | 1 | 1 |
|  | PBF highest quartile | 9.4(0.5) | 10.2(0.7) | 1.56(1.32,1.85) | 1.49(1.25,1.77) | 2.347(0.95,5.78) | 1.21(0.91,1.62) | 0.85(0.58,1.24) |
|  | Elevated BMI* | 8.9(0.4) | 19.1(0.8) | 2.55(2.25,2.88) | 2.57(2.24,2.94) | 6.10(2.94,12.63) | 2.33(1.88,2.89) | 2.23(1.56,3.19) |
|  | Both elevated BMI and PBF | 12.5(0.4) | 25.8(0.9) | 3.32(2.94,3.75) | 3.41(2.99,3.89) | 8.33(4.35,15.97) | 2.90(2.33,3.63) | 1.89(1.39,2.55) |
|  | *p interaction* |  |  | 0.25 | 0.90 | 0.35 | 0.89 | 0.99 |

Abbreviations: BMI, Body Mass Index; WC, waist circumference; PBF: percentage body fat

*Elevated WC: WC≥90 cm in male and WC≥80 cm in female

†Elevated BMI: BMI≥25 kg/m^2^

Data are presented as percentages (SE) or odds ratio (95% confidence interval).

Model 1: Adjusted for age.

Model 2: Adjusted for age, smoking (never smoker, current smoker, past smoker), alcohol consumption (non-drinker, mild to moderate drinker, heavy drinker), physical activity (regular exercise, non-regular exercise, no exercise), living with spouse or not, income (quartiles), educational attainment (≤ 6 years, 7-12 years, ≥13 years), energy intake from fat, and sodium consumption.
